# Bisphosphonate Use and Hospitalization for Hip Fractures in Women: An Observational Population-Based Study in France

**DOI:** 10.3390/ijerph18168780

**Published:** 2021-08-20

**Authors:** Bastien Bourrion, Cécile Souty, Lucie Fournier, Ana-Maria Vilcu, Thierry Blanchon, Pierre-Yves Böelle, Thomas Hanslik, Mathilde François

**Affiliations:** 1 Institut Pierre-Louis d’Epidémiologie et de Santé Publique (IPLESP UMRS 1136), INSERM, Sorbonne Université, 75012 Paris, France; cecile.souty@iplesp.upmc.fr (C.S.); luciefournier.90@gmail.com (L.F.); ana-maria.vilcu@iplesp.upmc.fr (A.-M.V.); thierry.blanchon@iplesp.upmc.fr (T.B.); py.boelle@gmail.com (P.-Y.B.); thomas.hanslik@aphp.fr (T.H.); dr.francois.m@gmail.com (M.F.); 2 Département de Médecine Générale, Faculté des Sciences de la Santé Simone Veil, Université Versailles-Saint-Quentin-en-Yvelines, 78180 Montigny le Bretonneux, France; 3 Service de Santé Publique, Hôpital Universitaire Saint-Antoine, AP-HP, 75012 Paris, France; 4 Service de Médecine Interne, Hôpital Universitaire Ambroise-Paré, AP-HP, 92100 Boulogne-Billancourt, France; 5 Faculté des Sciences de la Santé Simone Veil, Université de Versailles Saint-Quentin-en-Yvelines, 78180 Montigny-le-Bretonneux, France; 6 CESP, Bâtiment 15/16 Inserm, Hôpital Paul Brousse, 16 Avenue Paul Vaillant Couturier, INSERM, UVSQ, Université Paris-Saclay, 94807 Villejuif, France

**Keywords:** osteoporosis, hip fractures, bisphosphonates, hospitalization

## Abstract

Bisphosphonates are widely used in the treatment of women at risk of osteoporotic hip fracture; however, the overall effectiveness of bisphosphonates in the prevention of osteoporotic fractures has not been studied in real life. To investigate whether the use of bisphosphonates in women aged 50 years and over is associated with a decrease in hospitalization for osteoporotic hip fractures, a historical prospective cohort study was conducted between 2009 and 2016 from a permanent representative sample consisting of 1/97 of the French health insurance beneficiaries. Bisphosphonate use was defined according to medication persistence and adherence regarding bisphosphonate dispensations. The primary outcome was the hospitalization rate for osteoporotic hip fracture. Among the 81,268 women included, 2005 were exposed to bisphosphonates. The median time of bisphosphonate exposure was 12 (IQR, 3–29) and 17 (IQR, 5–42) months for the persistence and adherence definitions, respectively. Exposure to bisphosphonates was not associated with a decrease in hospitalization for hip fracture: weighted HRadherence = 0.66 (95% CI, 0.33 to 1.33); HRpersistance = 0.77 (95% CI, 0.38 to 1.57). In real life, bisphosphonate use does not appear to reduce hospitalization for hip fractures, as to date, it is probably prescribed as primary prevention and for a duration too short to be effective.

## 1. Introduction

In the 2000s, the worldwide incidence of osteoporotic fractures was estimated at 9 billion, of which 1.6 billion were hip fractures mainly occurring in women [1]. Among osteoporotic fractures, hip fractures have the strongest association with mortality [2]. The risk of death from any cause is six times higher for women after a hip fracture than for women without hip fracture. This risk decreases during the first two years after hip fracture, but it remains higher compared to the general population of the same age [2]. Age is an important risk factor for mortality after hip fracture, e.g., the risk of all-cause mortality in patients over 70 years of age is five to eight times higher in the first three months after a hip fracture [2]. The osteoporotic hip fracture is the only osteoporotic fracture that generally requires a hospital admission, which is the case for between 87% and 96% of patients [3].

The most prescribed therapeutic drug class for osteoporosis is bisphosphonates. Four bisphosphonates are available in France: zoledronic acid (intravenous administration), ibandronic acid (intravenous and oral administration), alendronic acid (oral administration) and risedronic acid (oral administration). The first clinical trials that allowed bisphosphonates to be marketed were evaluated in secondary prevention [4,5,6]. In these studies, risedronic acid was associated with a 39% (95% CI, 6% to 61%) reduction in the cumulative incidence of hip fracture over three years, and alendronic acid was associated with a 53% reduction in osteoporotic hip fracture at one year (95% CI, 24% to 74%) [4,5]; ibandronic acid has not shown effectiveness in reducing the risk of hip fracture [6]. The effectiveness of bisphosphonates in primary prevention for hip fracture has not been demonstrated [7,8]. A possible association between an increase in bisphosphonate use and a decrease in osteoporotic hip fractures has been demonstrated in aggregated data [9,10]. No population-based studies with individual longitudinal data have been carried out so far.

The objective of this population-based study using exhaustive administrative data was to investigate whether bisphosphonate use was associated with a reduction in the hospitalization rate for osteoporotic hip fracture.

## 2. Materials and Methods

### 2.1. Study Design and Setting

We conducted a historical prospective cohort study from 1 January 2009 to 31 December 2016, with prospective measurement of exposure using population-based French insurance health databases. We investigated a 1/97 random sample of all beneficiaries of the French health insurance called “échantillon généraliste de bénéficiaires” (EGB) [11]. The EGB constitutes a representative sample of the French population in terms of age, sex and geographical location, and it includes more than 660,000 individuals, whether they receive healthcare or not [11]. The EGB is an open cohort that is continuously updated with new beneficiaries and newborn infants and contains exhaustive information on all outpatient healthcare reimbursements. Data from individuals affiliated with the main French health insurance scheme (general scheme, covering 76% of the French population) have been stored since 2005, whereas data from individuals affiliated with other schemes have been available since 2011. The EGB is linked to the private and public hospital discharge database that contains dates of hospitalization and diagnoses (“Programme de médicalisation des systèmes d’information”, PMSI).

### 2.2. Participants

All women over 50 years old who were included in the database on 1 January 2009, i.e., who were covered by the general scheme, were eligible [12]. Women living outside metropolitan France were excluded because the risk of osteoporotic fracture depends on ethnicity [13]. To be included, a four-year period of historical data had to be available because the risk of recurrence of osteoporotic fracture remains significantly higher during the four years after a previous fracture [14]. Women with a history of metastatic cancer, renal failure, treatment for osteoporosis with medications other than bisphosphonates (i.e., teriparatide, raloxifene, denosumab or strontium ranelate) and treatment with hormone replacement therapy or antithyroid drugs were excluded [15,16]. Because residual bisphosphonates can remain for up to 18 months, women who received at least two bisphosphonate prescriptions in the two years before 1 January 2009 were excluded [5,17].

### 2.3. Exposure and Outcome

A bisphosphonate exposure consisted of at least two distinct bisphosphonate prescriptions (i.e., ibandronic acid, alendronic acid or risedronic acid). Women who received only one prescription were considered unexposed. Changes between bisphosphonate substances were allowed. To consider the potential bias between bisphosphonate prescription and use, two measures of bisphosphonate exposure were defined: persistence and adherence [18,19]. Persistence indicated that the patient stayed on therapy. It was defined as the time from initiation to the discontinuation of therapy [18,19]. We considered that a woman discontinued her therapy if the time between two consecutive prescriptions was at least three times the number of days covered by the supply dispensed. In this case, they were censored at the first prescription of this interval. Adherence indicated that a woman followed the treatment recommendations. We used the medication possession ratio (MPR), defined as the number of days covered by the supply obtained during the follow-up period divided by the number of days in this period. Ratios higher than 1 were systematically reduced to 1. Only women with an MPR over 0.8 were considered exposed and generally recognized as having good adherence [18]. Women with an MPR under 0.8 were considered unexposed.

The outcome was hospital admission for a cervical, trochanteric or subtrochanteric hip fracture, identified on discharge codes from the International Classification of Diseases, Tenth Revision (ICD-10): S72.0, S72.1 and S72.2.

Patients were censored after occurrence of the outcome, death, discontinuation of bisphosphonate treatment, zoledronic acid intake, prescription of an osteoporotic treatment other than bisphosphonates or prescription of hormone replacement.

### 2.4. Covariates

Patient comorbidities were ascertained based on ICD-10 codes extracted from the hospitalization records or from the records of patients’ long-term diseases available in the database [11]. Long-term diseases correspond to diseases in which the severity and/or the chronicity require a long-term and particularly costly treatment entirely covered by the health insurance. In addition, some illnesses have been identified by specific treatments, such as dementia, chronic pulmonary disease, human immunodeficiency virus (HIV), insulin-dependent diabetes and hyperthyroidism.

The Charlson Index [20] was computed at the start of the follow-up. All the covariates used in the Charlson Index and their weighting are described in Supplementary Material, Table S1.

Covariates known as osteoporotic fracture risk factors could be identified by the fracture risk assessment tool (FRAX) [21] (Supplementary Material, Table S2). As not all covariates composing the FRAX are available in our database, we included the following available covariates: age, history of hip fracture, corticosteroid intake (more than 5 mg per day within three months), history of rheumatoid arthritis, alcohol intake identified by diseases related to alcohol (Supplementary Material, Table S3 and history of secondary osteoporosis (Supplementary Material, Table S4).

Women with at least 1200 mg of calcium prescribed per day during the three years before the start of follow-up were considered treated. Similarly, women with at least 500 IU of vitamin D per day were considered treated [22,23].

### 2.5. Statistical analyses

Exposed and unexposed women were compared for continuous variables by Student’s t-test after checking for normal distribution or Wilcoxon’s test in case of non-equality of variances and Chi2 test or Fisher exact test for categorical variables.

The association between bisphosphonate use and hip fracture was studied using a marginal structural Cox model [24,25]. Standard methods for survival analysis, such as the time-dependent Cox model, may produce biased effect estimates when time-dependent confounders are themselves affected by previous treatment. The chosen model allows the creation of a pseudopopulation whose probability of exposure is independent of the covariates. In the unweighted analysis, this model was equivalent to an unadjusted and unweighted Cox model. Three types of analyses were carried out for adherence and persistence separately.

Unweighted analyses for each covariate to confirm the association between the outcome (hospitalization for hip fracture) and the covariate before inclusion in the weighted analysis.

Unweighted analyses to evaluate the association between the outcome and the bisphosphonate exposure defined as adherence or persistence, regardless of the bisphosphonate, and then for each bisphosphonate separately.

Weighted analyses to evaluate the association between the outcome and the bisphosphonate exposure defined as adherence or persistence, regardless of the bisphosphonate, and then for each bisphosphonate separately.

As recommended in a marginal structural Cox model, two types of variables have been defined for the analysis: non-time-dependent variables (the number of hip fractures before the follow-up, the Charlson index at the beginning of follow-up and calcium and vitamin D intake) and time-dependent variables (age, diabetes mellitus, malnutrition, chronic liver disease, rheumatoid arthritis, diseases related to alcohol and corticosteroid intake).

### 2.6. Sensitivity Analysis

Because the risk of hip fracture increases significantly after 75 years of age, we performed the same analyses considering only the subgroup of women aged 75 and older on 1 January 2009 [26].

Statistical analyses were performed using R, version 3.4.0 with the IPW package [27]. Significance testing was two-sided, and *p* < 0.05 was considered statistically significant.

### 2.7. Ethics

Access to these anonymous data is subject to prior training and authorization and has received approval from the French independent data protection administrative authority (CNIL).

## 3. Results

### 3.1. Characteristics of the Study Population

**Figure 1:**
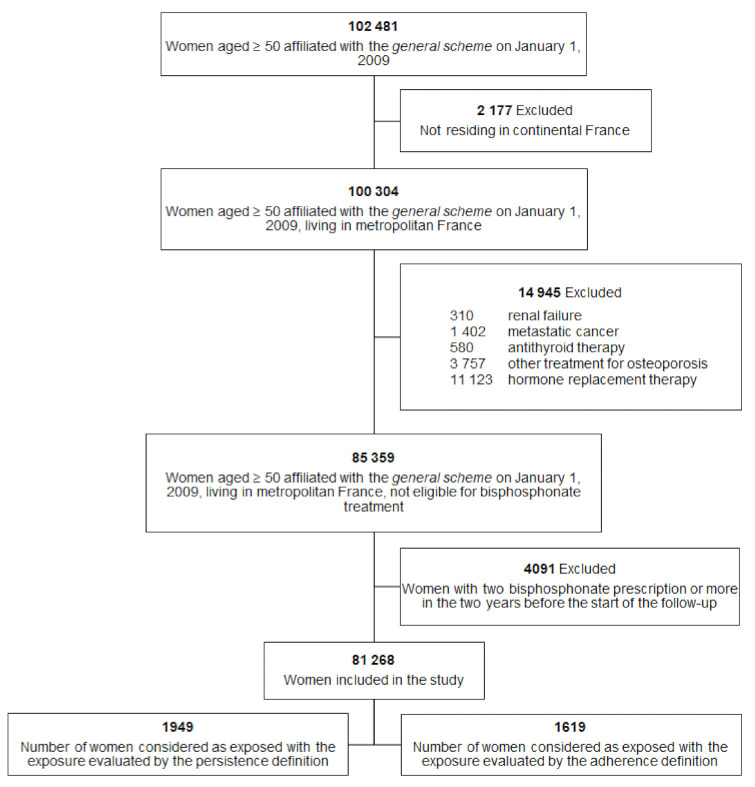
Study population.

On 1 January 2009, 102,481 women aged 50 years and older and who were covered by the general scheme were eligible. A total of 81,268 women (79.3%) were included in the study (Figure 1). Among these women, 2005 were exposed to bisphosphonates according to either the persistence or the adherence definition. The baseline characteristics of the women included are presented in Table 1. In this table, the interpretation of the *p*-value must be careful because of the large sample size. For example, the proportion of previous osteoporotic hip fracture is statistically higher in the exposed group but remains comparable (0.9 vs. 0.3).

The mean follow-up duration was 79.1 (95% CI, 78.9 to 79.4) and 79.4 (95% CI, 79.1 to 79.6) months, with a total of 535,820 and 537,496 person years in the persistence and adherence analyses, respectively. The median duration of bisphosphonate exposure was 12 (IQR, 3–29) and 17 (IQR, 5–42) months, respectively.

Between 2009 and 2016, the number of new hip fractures in the nonexposed women was 1160 (1.46%) versus 8 (0.41%) in the exposed women according to persistence analyses and 1169 (1.47%) versus 8 (0.49%) according to adherence analyses. The mean age of hip fracture was 81 years (95% CI, 80.6 to 81.7).

In the study population, 22,814 women were aged 75 years and older. The number of new hip fractures in the nonexposed women was 793 and 801 in the persistence and adherence analyses, respectively, versus 8 in the exposed women in both analyses. The mean follow-up duration was 52.7 (95% CI, 52.1 to 53.3) and 52.9 months (95% CI, 52.3 to 53.5) in the persistence and adherence analyses, respectively.

### 3.2. Covariates Analyses

The risk of hip fracture was strongly associated with a past history of hip fracture, with an HR of 4.85 (95% CI, 3.45 to 6.82) (Table 2). Except for rheumatoid arthritis, other diseases associated with secondary osteoporosis were statistically associated with the risk of hip fracture.

### 3.3. Association between Hospitalization for Hip Fracture and Bisphosphonate Use

In the unweighted analyses, the association between hospitalization for hip fracture and the bisphosphonate exposure defined as either adherence or persistence was not significant (Table 3). In the weighted analyses, the association remained nonsignificant, with a Hazard ratio (HR) of 0.77 (95% CI, 0.38 to 1.56) and 0.66 (95% CI, 0.33 to 1.33), respectively, for persistence and adherence analyses. Analyses of bisphosphonate substance had the same results (Table 4).

Weighted and unweighted analyses for women aged 75 and older (Table 3) also displayed no significant associations between exposure and osteoporotic hip fracture, regardless of the exposure definition considered.

## 4. Discussion

To our knowledge, this is the largest historical prospective cohort study that investigates the association between the exposure to bisphosphonates and hospitalization for osteoporotic hip fracture. We found that exposure to bisphosphonates was not associated with a decrease in hospitalization for osteoporotic hip fracture for women aged 50 years and older. This result is reinforced by the sensitivity analyses for women aged 75 and older who also displayed no significant associations.

Since the commercialization of bisphosphonates, their efficacy has been regularly analysed. A recent meta-analysis reported no association between the risk of hip fracture and bisphosphonate treatment [28]. In contrast, another recent meta-analysis concluded that alendronic acid and risedronic acid could reduce the hip fracture risk [29]. Although these reviews use the same method of analysis, they differ in the articles found in the literature and included after measuring the risk of bias.

In our study, the mean age of the exposed women was 67 (IQR, 59 to 77) years, which was younger than the mean age of hip fracture (81 years). This suggests that bisphosphonates were prescribed for primary prevention that was not consistent with the indication for bisphosphonates [30]. In this context, our results are consistent with two meta-analyses that showed the effectiveness of alendronic acid and risedronic acid only in the secondary prevention of osteoporotic fractures [7,8].

Another explanation of the lack of efficacy of bisphosphonates in this study comes from the short duration of bisphosphonate consumption (of 12 and 17 months in the persistence and adherence analyses, respectively) [31]. Indeed, the time to benefit (TTB) of bisphosphonates, defined as the estimated time it takes for a bisphosphonate treatment to become significantly effective in a group of patients, is relatively long. For example, the TTB of alendronate is estimated to be 8 months for patients over 70 years of age and 19 months for patients under 70 years of age [32]. Thus, we could have done an analysis of women considered correctly treated versus women considered incorrectly treated. Unfortunately, the time to benefit is only known for Alendronate in secondary prevention. It is therefore likely to be longer in primary prevention, but unknown. Thus, as there is no defined time to compare correctly treated and incorrectly treated patients, this analysis is not really feasible.

However, there are other interventions that can reduce the risk of osteoporotic fracture. A meta-analysis showed that exercise programs can significantly reduce falls and hip fractures (OR = 0.39; 95% CI, 0.22 to 0.66) [33]. Similarly, cataract surgery significantly decreased the hip fracture risk in the year after a fall (OR = 0.84; 95% CI, 0.81 to 0.87) [34]. Unfortunately, there are no studies comparing these interventions to the use of bisphosphonates.

### Strengths and Limitations

The main strength of this study was the large population size. This allowed us to identify a large number of hip fractures, of which the incidence is low in the population. Fractures were identified by the following ICD-10 codes: S72.0, S72.1 and S72.2. These codes slightly overestimated patient hospitalization with osteoporotic hip fractures by 4.6% (95% CI, 3.8% to 5.1%) [35,36], which results in a nondifferential classification bias, as the exposure was overestimated in the exposed and unexposed groups. Second, we used a marginal structural Cox model that allowed us to control for an indication bias, the main issue in an observational study. As some variables vary over time, conventional approaches using adjustment would have been biased. The unweighted models showed a nonsignificant lower proportion of fractures in the treated patient group. This result was not found with the weighted models. This could be explained by an indication bias: patients in poor health with a high fracture risk are probably treated less than others because of their many other health problems. The use of a marginal structural Cox model has corrected this bias. The third strength of this study was the use of the Charlson Index, which allowed us to estimate the patients’ health status. We also used FRAX variables to identify risk factors for hip fracture. This allowed us to control for confounding factors related to the risk of osteoporotic fracture.

The main limitations are related to the administrative medical databases. First, the EGB contains data about drug prescriptions and not information on real use. To limit this bias, we considered women as exposed if they filled two prescriptions. Second, we could not consider zoledronic acid (administered only intravenously) because data about this treatment were not available in the database. Third, for outpatients, diagnoses were not collected by the health insurance system. Ascertainment of chronic conditions was based on the registered patient’s long-term diseases, reimbursements for disease-specific treatments and hospitalization diagnoses. Fourth, we did not study bisphosphonate side effects because they do not require systematic hospitalization or result in long-term diseases.

## 5. Conclusions

In real life, bisphosphonate use does not appear to reduce hospitalization for osteoporotic hip fractures, as to date, it is probably prescribed as primary prevention and for a duration too short to be effective. To evaluate the benefit–risk balance of the treatment, it would also be necessary to take into account the side effects that we were unable to study. Further studies to understand the reasons for inappropriately short prescriptions are needed: is it a problem of tolerance or of compliance with indications by doctors? Information of TTB should be shared with patients before any decision to treat is made.

## Figures and Tables

**Table 1:** Baseline characteristics of women included on 1 January 2009.

Characteristics	Nonexposed (*N* = 79,263)	Exposed (*N* = 2005)	*p* Value ^a^
Age, median (IQR), y	64 (56–76)	67 (59–75)	<0.001
History of hip fracture, No. (%)	720 (0.9)	7 (0.3)	<0.001
Insulin-dependent diabetes, No. (%)	496 (0.6)	11 (0.5)	0.66
Malnutrition, No. (%)	26 (0.0)	1 (0.0)	0.49
Alcohol-related diseases, No. (%)	323 (0.4)	9 (0.4)	0.77
Osteogenesis imperfecta, No. (%)	0 (0.0)	0 (0.0)	- ^b^
Hypogonadism, No. (%)	1 (0.0)	0 (0.0)	-
Rheumatoid arthritis, No. (%)	225 (0.3)	9 (0.4)	0.17
Charlson index, median (IQR)	0 (0–0)	0 (0–1)	0.07
Chronic liver disease, No. (%)	193 (0.2)	6 (0.3)	0.64
Ischemic heart disease, No. (%)	1329 (1.7)	28 (1.4)	0.33
Congestive heart failure, No. (%)	923 (1.2)	8 (0.4)	0.001
Diseases of arterioles and capillaries, No. (%)	586 (0.7)	16 (0.8)	0.76
Cerebrovascular disease, No. (%)	889 (1.1)	13 (0.6)	0.05
Dementia, No. (%)	1605 (2.0)	9 (0.4)	<0.001
Chronic pulmonary disease, No. (%)	10,468 (13.2)	357 (17.8)	<0.001
Systemic connective tissue disorders, No. (%)	133 (0.2)	11 (0.5)	<0.001
Peptic ulcer disease, No. (%)	211 (0.3)	4 (0.2)	0.82
Diabetes without organ damage, No. (%)	6159 (7.8)	97 (4.8)	<0.001
Diabetes with organ damage, No. (%)	0 (0.0)	0 (0.0)	-
Hemiplegia, No. (%)	105 (0.1)	2 (0.1)	>0.99
Renal failure, No. (%)	0 (0.0)	0 (0.0)	-
Metastatic tumor, No. (%)	0 (0.0)	0 (0.0)	-
Tumor without metastasis, No. (%)	2650 (3.3)	104 (5.2)	<0.001
Lymphoma or leukemia, No. (%)	206 (0.3)	9 (0.4)	0.10
HIV, No. (%)	37 (0.0)	2 (0.1)	0.25
Polymyalgia rheumatica, No. (%)	17 (0.0)	1 (0.0)	0.36
Corticosteroids, median (IQR), mg	40 (15–44)	40 (18–69)	0.20
Calcium, No. (%)	504 (0.6)	43 (2.1)	<0.001
Vitamin D, No. (%)	2795 (3.5)	161 (8.0)	<0.001

Abbreviations: IQR, interquartile range; mg, milligram. ^a^ according to the variables, we used Wilcoxon test, Chi2 test or Fisher test. ^b^ not computable.

**Table 2:** Association between hospitalization for a hip fracture and the covariates: unweighted analyses.

Characteristics	HR	95% CI	*p* Value
History of hip fracture ^a^	4.85	3.45–6.82	<0.001
Charlson index ^a^	1.56	1.49–1.64	<0.001
Age ^b^	1.08	1.08–1.08	<0.001
Insulin-dependent diabetes ^b^	2.83	1.75–4.57	<0.001
Malnutrition ^b^	7.14	3.20–15.94	<0.001
Chronic liver disease ^b^	2.16	1.16–4.02	0.02
Rheumatoid arthritis ^b^	1.18	0.61–2.28	0.61
Alcohol-related diseases ^b^	2.45	1.56–3.86	<0.001
Corticosteroids ^b^	1.00	1.00–1.00	<0.001
Calcium ^a^	1.84	1.07–3.18	0.03
Vitamin D ^a^	2.48	2.00–3.07	<0.001

HR, Hazard ratio. CI, confidence interval. ^a^ start of follow-up. ^b^ time-dependent.

**Table 3:** Risk of hospitalization for hip fracture after bisphosphonate consumption or no bisphosphonate consumption according to the persistence and adherence definitions.

		Persistence		Adherence	
		HR_E/NE_	95% CI	HR_E/NE_	95% CI
Main analysesAge ≥ 50 years	Unweighted	1.11	0.55–2.22	0.97	0.49–1.95
	Weighted	0.77	0.38–1.56	0.66	0.33–1.33
Sensitivity analysisAge ≥ 75 years	Unweighted	1.16	0.58–2.33	1.03	0.51–2.07
	Weighted	1.39	0.69–2.83	1.31	0.65–2.64

Abbreviation: HRE/NE, Hazard ratio for exposed versus nonexposed; CI, confidence interval.

**Table 4:** Risk of hospitalization for hip fracture after bisphosphonate consumption or no bisphosphonate consumption according to the persistence and adherence definitions and the bisphosphonate type.

		Persistence		Adherence	
		HR_E/NE_	95% CI	HR_E/NE_	95% CI
**Alendronic acid**	Unweighted	0.66	0.09–4.69	1.60	0.60–4.28
	Weighted	0.41	0.06–2.90	0.41	0.06–2.90
**Ibandronic acid**	Unweighted	3.21	0.80–12.90	2.77	0.69–11.16
	Weighted	1.99	0.47–8.35	1.97	0.47–8.35
**Risedronic acid**	Unweighted	3.21	0.80–12.90	2.77	0.69–11.16
	Weighted	1.99	0.47–8.35	1.97	0.47–8.35

Abbreviation: HRE/NE, Hazard ratio for exposed versus nonexposed; CI, confidence interval.

## Data Availability

EGB data are not available.

## Supplementary Materials

The following are available online at https://www.mdpi.com/article/10.3390/ijerph18168780/s1, Table S1: Definition of the Charlson Comorbidity Index, Table S2: Variables of the fracture risk assessment tool, Table S3: Definition of alcohol intake identified by disease related to alcohol, Table S4: Definition of diseases related to secondary osteoporosis.
