# Supplementary material for: Bisphosphonate Use and Hospitalization for Hip Fractures in Women: An Observational Population-Based Study in France
Source: Int J Environ Res Public Health. 2021 Aug 20;18(16):8780. doi: 10.3390/ijerph18168780 (PMC8392579; doi:10.3390/ijerph18168780)
Supplement: Supplementary file 1 [file ijerph-18-08780-s001.zip › ijerph-1298380-supplementary.pdf]

Supplementary file, Table S1: Definition of the Charlson comorbidity index

ICD-10	Description	Associated treatment	Weight
I20 - I25	Ischemic heart disease	NU[a]	1
I50	Congestive heart failure	NU[a]	1
I70 - I79 (no I781)	Diseases of arterioles and capillaries	NU[a]	1
I60 - I69	Cerebrovascular disease	NU[a]	1
F00 - F03	Dementia	donepezil, galantamine, memantine, rivastigmine	1
J43 - J46	Chronic pulmonary diseases	β_2-agonist, inhaled corticosteroids, muscarinic antagonists	1
M30 - M36 (no M353)	Systemic connective tissue disorders	NU[a]	1
K25 - K27	Peptic ulcer disease	NU[a]	1
E10 - E14 (no diabetic code with organ damage)	Diabetes without organ damage	NU[a]	1
G81	Hemiplegia	NU[a]	2
N17 - N19	Renal failure	NU[a]	2
E102 - 105, E107, E112 - E115, E117, H360, H360A, H360B, H360X	Diabetes with organ damage	NU[a]	2
C77 - C79	Tumor without metastasis	NU[a]	2
C81 - C96	Lymphoma or leukemia	NU[a]	2
K70 - K77	Chronic liver disease	NU[a]	3
C77 - C79	Metastatic tumor	NU[a]	6
B20-B22, B24, Z21	HIV	HAART	6

Abbreviation: HAART, Highly active antiretroviral therapy

[a] associated treatments were not used (NU)

Supplementary file, Table S2: Variables of the fracture risk assessment tool

Variables	
Age	40 to 90 years
Sex	
Weight	
Height	
History of osteoporotic fracture	
Mother and father history of hip fracture	
Tobacco	
Corticosteroid intake	More than 5 mg per day within 3 months
Rheumatoid arthritis	
Secondary osteoporosis (Online Resource Table 4)	
Insulin-dependent diabetes	
Osteogenesis imperfecta	
Untreated hyperthyroidism	
Hypogonadism	
Menopause before age 45 years	
Malnutrition	
Chronic liver disease	
Alcohol intake (Online Resource Table 3)	More than 3 units of alcohol per day
Bone mineral density	

Supplementary file, Table S3: Definition of alcohol intake identified by disease related to alcohol

ICD-10	Description	Associated treatment
F10	Mental and behavioral disorders due to use of alcohol	NU
G312	Degeneration of the nervous system due to alcohol	NU
G621	Alcoholic polyneuropathy	NU
G721	Alcoholic myopathy	NU
I426	Alcoholic cardiomyopathy	NU
K292	Alcoholic gastritis	NU
K70	Alcoholic liver disease	NU
K852	Alcohol-induced acute pancreatitis	NU
K860	Alcohol-induced chronic pancreatitis	NU
T51	Toxic effect of alcohol	NU
Y912	Severe alcohol intoxication	NU
Y913	Very severe alcohol intoxication	NU

Abbreviation: NU, associated treatments were not used

Supplementary file, Table S4: Definition of diseases related to secondary osteoporosis

ICD-10	Description	Associated treatment
E10	Insulin-dependent diabetes	Insulin consumption without history of oral treatment
Q780	Osteogenesis imperfecta	NU
E28 - E29 (no: E280, E290)	Hypogonadism	NU
E40 - E46	Malnutrition	NU
E05	Hyperthyroidism	benzyl thiouracil, carbimazole, propylthiouracil, thiamazole
K70 - K77 (no: K701, K71, K710, K711, K712, K716, K751, K750)	Chronic liver disease	NU

Abbreviation: NU, associated treatments were not used
